# [5-(2-Fur­yl)-6-nitro-1,2,3,5,6,7-hexa­hydro­imidazo[1,2-*a*]pyridin-8-yl](phen­yl)methanone

**DOI:** 10.1107/S1600536809033716

**Published:** 2009-08-29

**Authors:** Muhammad Yaqub, Zahid Shafiq, Ashfaq M. Qureshi, Muhammad Najam-ul-Haq

**Affiliations:** a Department of Chemistry, Bahauddin Zakariya University, Multan 60800, Pakistan

## Abstract

In the title compound, C_18_H_17_N_3_O_4_, the furyl and phenyl rings are inclined at almost right angles [85.77 (7) and 63.25 (7)°, respectively] to the central imidazo[1,2-*a*]pyridinyl unit. The structure displays both inter- and intra­molecular N—H⋯O hydrogen bonding.

## Related literature

For cyclic 1,1-enediamines as inter­mediates for the construction of heterocyclic compounds, see: Yu *et al.* (2006 ▶); Yaqub *et al.* (2008 ▶); Wang *et al.* (1999 ▶). For related structures, see: Yu *et al.* (2007 ▶); Yaqub *et al.* (2009 ▶).
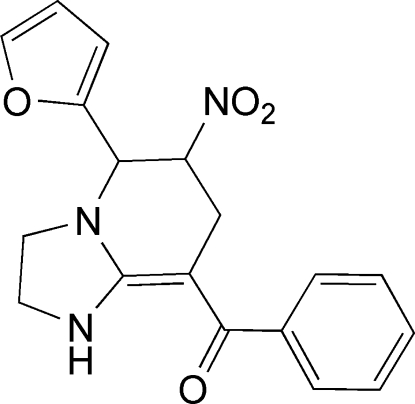


## Experimental

### 

#### Crystal data

C_18_H_17_N_3_O_4_
*M* *_r_* = 339.35
Monoclinic, 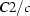

*a* = 11.980 (2) Å
*b* = 15.047 (3) Å
*c* = 17.932 (4) Å
β = 101.94 (3)°
*V* = 3162.7 (11) Å^3^
*Z* = 8
Mo *K*α radiation
μ = 0.10 mm^−1^
*T* = 173 K
0.45 × 0.33 × 0.32 mm

#### Data collection

Rigaku R-AXIS RAPID IP area-detector diffractometer
Absorption correction: multi-scan (*ABSCOR*; Higashi, 1995 ▶) *T*_min_ = 0.955, *T*_max_ = 0.968
5401 measured reflections
2801 independent reflections
2426 reflections with *I* > 2σ(*I*)
*R*_int_ = 0.031

#### Refinement

*R*[*F* ^2^ > 2σ(*F* ^2^)] = 0.055
*wR*(*F* ^2^) = 0.128
*S* = 1.14
2801 reflections
226 parameters
H-atom parameters constrained
Δρ_max_ = 0.37 e Å^−3^
Δρ_min_ = −0.36 e Å^−3^

### 

Data collection: *RAPID-AUTO* (Rigaku, 2001 ▶); cell refinement: *RAPID-AUTO*; data reduction: *RAPID-AUTO*; program(s) used to solve structure: *SHELXS97* (Sheldrick, 2008 ▶); program(s) used to refine structure: *SHELXL97* (Sheldrick, 2008 ▶); molecular graphics: *XP* in *SHELXTL* (Sheldrick, 2008 ▶); software used to prepare material for publication: *SHELXL97*.

## Supplementary Material

Crystal structure: contains datablocks I, global. DOI: 10.1107/S1600536809033716/pv2191sup1.cif

Structure factors: contains datablocks I. DOI: 10.1107/S1600536809033716/pv2191Isup2.hkl

Additional supplementary materials:  crystallographic information; 3D view; checkCIF report

## Figures and Tables

**Table 1:** Hydrogen-bond geometry (Å, °)

*D*—H⋯*A*	*D*—H	H⋯*A*	*D*⋯*A*	*D*—H⋯*A*
N1—H1*A*⋯O4	0.88	2.17	2.714 (2)	119
N1—H1*A*⋯O4^i^	0.88	2.40	3.025 (3)	128

Symmetry code: (i) 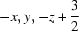.

**Fig. 1.**
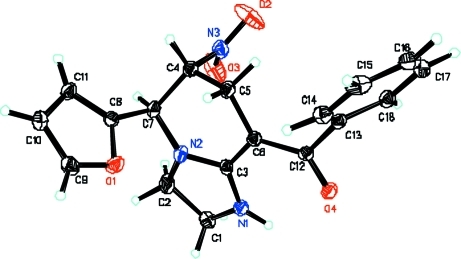
The molecular structure of the title compound.

**Fig. 2.**
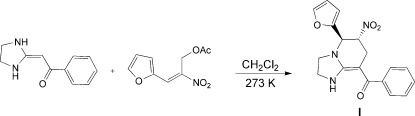
The formation of the title compound.

## supplementary crystallographic information

### Comment

The significance of cyclic 1,1-enediamines known as heterocyclic ketene aminals (HKAs) is obvious as a building block in many synthetic operations especially as intermediates for the construction of novel heterocyclic compound (Yaqub *et al.*, 2008; Yu  *et al.*, 2006). The condensation through *β*-carbon and secondary nitrogen with bis-electrophile to form heterocyclic ring is one of the most favorable features of HKAs, (Yaqub *et al.*, 2009; Yu, *et al.*, 2007; Wang *et al.*, 1999) which was utilized to synthesize the title compound (I) by treating nitro derivative of Baylis-Hillman acetates with five membered analogue of heterocyclic ketene aminal. In this paper, we describe the crystal structure of (I).

In the title compound (Fig. 1), the pyridyl ring of the nitroimidazo-pyridinyl moiety adopts a half-chair conformation with C4 lying 0.666 (3) Å out of the plane formed by the atoms N1/N2/C1/C2/C3/C5/C6/C7; N2 showing the maximum deviation of 0.069 (2) Å from this plane. The mean-planes of the furanyl and benzyl rings lie at angles 85.77 (7) and 0.63.25 (7)°, respectively, with respect to the mean-plane formed by the atoms N1/N2/C1/C2/C3/C5/C6/C7 of the nitroimidazo-pyridinyl moiety. The structure is stabilized by both inter- and intra-molecular hydrogen bonding of the type N—H···O (details have been provided in Table 1).

### Experimental

Heterocyclic ketene aminal (0.13 g, 0.71 mmol) and (*E*)-2-nitro-3-(2-furanyl)allylacetate (0.15 g, 0.71 mmol) were stirred in 30 ml of dichloromethane at 273 K for one hour, followed by stirring at room temperature for 6 h (Scheme 2). Solvent was evaporated and residue was passed through short chromatographic column. The elution was carried out by petroleum ether: ethyl acetate (4:1) mixture to get the title compound (I) as a light yellow solid. The single crystals were grown in dichloromethane - petroleum ether (1:5) system at room temperature by slow evaporation. Yield: 75% (0.18 g), m.p. 440–441 K (lit. m. p. 441–442 K) (Yaqub *et al.*, 2008).

### Refinement

The H atoms were positioned geometrically and refined in riding mode, with N–H = 0.88 Å, C—H = 0.95, 0.99 and 1.00 Å, for aryl, methylene and methine type H-atoms and *U*_iso_(H) = 1.2 *U*_eq_(parent atoms).

### Crystal data

**Table:**

C_18_H_17_N_3_O_4_	*F*(000) = 1424
*M**_r_* = 339.35	*D*_x_ = 1.425 Mg m^−^^3^
Monoclinic, *C*2/*c*	Mo *K*α radiation, λ = 0.71073 Å
Hall symbol: -C 2yc	Cell parameters from 989 reflections
*a* = 11.980 (2) Å	θ = 2.2–27.5°
*b* = 15.047 (3) Å	µ = 0.10 mm^−^^1^
*c* = 17.932 (4) Å	*T* = 173 K
β = 101.94 (3)°	Block, yellow
*V* = 3162.7 (11) Å^3^	0.45 × 0.33 × 0.32 mm
*Z* = 8	

### Data collection

**Table:**

Rigaku R-AXIS RAPID IP area-detector diffractometer	2801 independent reflections
Radiation source: rotating anode	2426 reflections with *I* > 2σ(*I*)
graphite	*R*_int_ = 0.031
ω scans at fixed χ = 45°	θ_max_ = 25.0°, θ_min_ = 2.2°
Absorption correction: multi-scan (*ABSCOR*; Higashi, 1995)	*h* = −14→14
*T*_min_ = 0.955, *T*_max_ = 0.968	*k* = −17→17
5401 measured reflections	*l* = −21→21

### Refinement

**Table:**

Refinement on *F*^2^	Primary atom site location: structure-invariant direct methods
Least-squares matrix: full	Secondary atom site location: difference Fourier map
*R*[*F*^2^ > 2σ(*F*^2^)] = 0.055	Hydrogen site location: inferred from neighbouring sites
*wR*(*F*^2^) = 0.128	H-atom parameters constrained
*S* = 1.14	*w* = 1/[σ^2^(*F*_o_^2^) + (0.0499*P*)^2^ + 3.4672*P*] where *P* = (*F*_o_^2^ + 2*F*_c_^2^)/3
2801 reflections	(Δ/σ)_max_ < 0.001
226 parameters	Δρ_max_ = 0.37 e Å^−^^3^
0 restraints	Δρ_min_ = −0.36 e Å^−^^3^

### Special details

**Table:**

Geometry. All e.s.d.'s (except the e.s.d. in the dihedral angle between two l.s. planes) are estimated using the full covariance matrix. The cell e.s.d.'s are taken into account individually in the estimation of e.s.d.'s in distances, angles and torsion angles; correlations between e.s.d.'s in cell parameters are only used when they are defined by crystal symmetry. An approximate (isotropic) treatment of cell e.s.d.'s is used for estimating e.s.d.'s involving l.s. planes.
Refinement. Refinement of *F*^2^ against ALL reflections. The weighted *R*-factor *wR* and goodness of fit *S* are based on *F*^2^, conventional *R*-factors *R* are based on *F*, with *F* set to zero for negative *F*^2^. The threshold expression of *F*^2^ > σ(*F*^2^) is used only for calculating *R*-factors(gt) *etc*. and is not relevant to the choice of reflections for refinement. *R*-factors based on *F*^2^ are statistically about twice as large as those based on *F*, and *R*- factors based on ALL data will be even larger.

### Fractional atomic coordinates and isotropic or equivalent isotropic displacement parameters (Å^2^)

**Table:**

	*x*	*y*	*z*	*U*_iso_*/*U*_eq_	
O1	0.43109 (14)	0.20626 (10)	0.91996 (10)	0.0384 (4)	
O2	0.2016 (2)	0.3319 (2)	0.63070 (12)	0.0835 (8)	
O3	0.12099 (18)	0.35426 (18)	0.72302 (12)	0.0722 (7)	
O4	0.11567 (13)	0.00988 (10)	0.71141 (9)	0.0319 (4)	
N1	0.10262 (16)	0.11596 (13)	0.83171 (10)	0.0327 (5)	
H1A	0.0780	0.0626	0.8163	0.039*	
N2	0.21072 (15)	0.23408 (12)	0.84736 (11)	0.0302 (5)	
N3	0.20184 (17)	0.32982 (14)	0.69714 (12)	0.0370 (5)	
C1	0.05883 (19)	0.16761 (16)	0.88815 (13)	0.0329 (5)	
H1B	0.0652	0.1341	0.9363	0.039*	
H1C	−0.0219	0.1844	0.8689	0.039*	
C2	0.1352 (2)	0.2487 (2)	0.89931 (15)	0.0473 (7)	
H2A	0.0902	0.3038	0.8863	0.057*	
H2B	0.1785	0.2527	0.9526	0.057*	
C3	0.18456 (17)	0.15910 (14)	0.80613 (12)	0.0244 (5)	
C7	0.29353 (18)	0.29889 (14)	0.83329 (13)	0.0280 (5)	
H7A	0.2646	0.3596	0.8419	0.034*	
C4	0.30665 (18)	0.29262 (15)	0.75038 (13)	0.0289 (5)	
H4A	0.3742	0.3290	0.7446	0.035*	
C5	0.32660 (18)	0.19731 (14)	0.72796 (13)	0.0286 (5)	
H5A	0.3265	0.1949	0.6728	0.034*	
H5B	0.4024	0.1773	0.7560	0.034*	
C6	0.23556 (17)	0.13503 (14)	0.74538 (12)	0.0247 (5)	
C8	0.40885 (18)	0.28698 (14)	0.88525 (12)	0.0254 (5)	
C9	0.5402 (2)	0.21114 (16)	0.96169 (14)	0.0359 (6)	
H9A	0.5781	0.1644	0.9925	0.043*	
C10	0.58593 (19)	0.28997 (15)	0.95339 (13)	0.0312 (5)	
H10A	0.6607	0.3094	0.9761	0.037*	
C11	0.50011 (19)	0.33953 (15)	0.90356 (13)	0.0318 (5)	
H11A	0.5067	0.3987	0.8866	0.038*	
C12	0.19701 (17)	0.05876 (14)	0.70164 (12)	0.0240 (5)	
C13	0.25482 (18)	0.03347 (13)	0.63770 (12)	0.0243 (5)	
C14	0.37074 (19)	0.01371 (15)	0.65132 (14)	0.0327 (5)	
H14A	0.4159	0.0210	0.7011	0.039*	
C15	0.4210 (2)	−0.01643 (16)	0.59321 (16)	0.0402 (6)	
H15A	0.4997	−0.0314	0.6036	0.048*	
C16	0.3569 (2)	−0.02476 (15)	0.52020 (15)	0.0396 (6)	
H16A	0.3916	−0.0449	0.4802	0.047*	
C17	0.2425 (2)	−0.00372 (16)	0.50545 (14)	0.0360 (6)	
H17A	0.1987	−0.0080	0.4549	0.043*	
C18	0.19096 (19)	0.02370 (14)	0.56414 (13)	0.0296 (5)	
H18A	0.1114	0.0359	0.5539	0.036*	

### Atomic displacement parameters (Å^2^)

**Table:**

	*U*^11^	*U*^22^	*U*^33^	*U*^12^	*U*^13^	*U*^23^
O1	0.0377 (9)	0.0257 (8)	0.0469 (11)	−0.0042 (7)	−0.0025 (8)	0.0091 (7)
O2	0.0725 (15)	0.145 (2)	0.0326 (12)	0.0368 (16)	0.0095 (10)	0.0255 (13)
O3	0.0543 (13)	0.109 (2)	0.0460 (13)	0.0391 (13)	−0.0055 (10)	−0.0096 (12)
O4	0.0336 (9)	0.0286 (8)	0.0354 (9)	−0.0075 (7)	0.0116 (7)	−0.0056 (7)
N1	0.0386 (11)	0.0359 (11)	0.0264 (10)	−0.0111 (9)	0.0133 (9)	−0.0063 (8)
N2	0.0266 (10)	0.0309 (10)	0.0345 (11)	−0.0049 (8)	0.0098 (8)	−0.0099 (8)
N3	0.0362 (11)	0.0383 (12)	0.0333 (12)	−0.0004 (9)	−0.0006 (9)	−0.0010 (9)
C1	0.0270 (11)	0.0424 (13)	0.0301 (12)	0.0030 (10)	0.0077 (9)	−0.0023 (10)
C2	0.0517 (16)	0.0595 (18)	0.0352 (15)	−0.0184 (14)	0.0196 (12)	−0.0203 (13)
C3	0.0224 (10)	0.0259 (11)	0.0223 (11)	0.0008 (9)	−0.0013 (8)	−0.0001 (9)
C7	0.0284 (11)	0.0224 (11)	0.0315 (12)	−0.0015 (9)	0.0021 (9)	−0.0052 (9)
C4	0.0260 (11)	0.0277 (12)	0.0309 (12)	−0.0036 (9)	0.0010 (9)	−0.0002 (9)
C5	0.0269 (11)	0.0302 (12)	0.0294 (12)	−0.0043 (9)	0.0069 (9)	−0.0038 (9)
C6	0.0229 (10)	0.0274 (11)	0.0236 (11)	−0.0023 (8)	0.0042 (9)	−0.0018 (9)
C8	0.0313 (12)	0.0212 (10)	0.0239 (11)	0.0008 (9)	0.0064 (9)	−0.0007 (9)
C9	0.0341 (13)	0.0347 (13)	0.0341 (14)	0.0052 (10)	−0.0038 (10)	0.0043 (10)
C10	0.0277 (12)	0.0322 (12)	0.0314 (13)	0.0004 (9)	0.0010 (10)	−0.0056 (10)
C11	0.0330 (12)	0.0226 (11)	0.0373 (13)	−0.0044 (9)	0.0013 (10)	0.0009 (10)
C12	0.0232 (11)	0.0233 (11)	0.0247 (11)	0.0016 (8)	0.0032 (9)	0.0025 (9)
C13	0.0285 (11)	0.0174 (10)	0.0278 (12)	−0.0002 (8)	0.0078 (9)	−0.0013 (8)
C14	0.0286 (12)	0.0327 (12)	0.0360 (13)	0.0024 (10)	0.0049 (10)	0.0049 (10)
C15	0.0364 (13)	0.0335 (13)	0.0562 (17)	0.0082 (11)	0.0226 (13)	0.0089 (12)
C16	0.0561 (16)	0.0248 (12)	0.0469 (16)	0.0038 (11)	0.0316 (13)	−0.0007 (11)
C17	0.0514 (15)	0.0284 (12)	0.0297 (13)	−0.0041 (11)	0.0118 (11)	−0.0044 (10)
C18	0.0314 (12)	0.0253 (11)	0.0313 (13)	0.0001 (9)	0.0048 (10)	−0.0014 (9)

### Geometric parameters (Å, °)

**Table:**

O1—C8	1.366 (3)	C5—C6	1.519 (3)
O1—C9	1.367 (3)	C5—H5A	0.9900
O2—N3	1.191 (3)	C5—H5B	0.9900
O3—N3	1.214 (3)	C6—C12	1.413 (3)
O4—C12	1.262 (3)	C8—C11	1.334 (3)
N1—C3	1.335 (3)	C9—C10	1.328 (3)
N1—C1	1.457 (3)	C9—H9A	0.9500
N1—H1A	0.8800	C10—C11	1.425 (3)
N2—C3	1.350 (3)	C10—H10A	0.9500
N2—C2	1.443 (3)	C11—H11A	0.9500
N2—C7	1.450 (3)	C12—C13	1.506 (3)
N3—C4	1.518 (3)	C13—C18	1.389 (3)
C1—C2	1.513 (3)	C13—C14	1.392 (3)
C1—H1B	0.9900	C14—C15	1.384 (3)
C1—H1C	0.9900	C14—H14A	0.9500
C2—H2A	0.9900	C15—C16	1.380 (4)
C2—H2B	0.9900	C15—H15A	0.9500
C3—C6	1.402 (3)	C16—C17	1.378 (4)
C7—C8	1.509 (3)	C16—H16A	0.9500
C7—C4	1.530 (3)	C17—C18	1.389 (3)
C7—H7A	1.0000	C17—H17A	0.9500
C4—C5	1.522 (3)	C18—H18A	0.9500
C4—H4A	1.0000		
			
C8—O1—C9	106.02 (17)	C6—C5—H5B	109.3
C3—N1—C1	112.05 (19)	C4—C5—H5B	109.3
C3—N1—H1A	124.0	H5A—C5—H5B	108.0
C1—N1—H1A	124.0	C3—C6—C12	119.75 (19)
C3—N2—C2	112.04 (18)	C3—C6—C5	116.57 (19)
C3—N2—C7	123.81 (18)	C12—C6—C5	123.59 (19)
C2—N2—C7	123.56 (19)	C11—C8—O1	110.01 (19)
O2—N3—O3	122.5 (2)	C11—C8—C7	132.9 (2)
O2—N3—C4	117.9 (2)	O1—C8—C7	117.03 (18)
O3—N3—C4	119.5 (2)	C10—C9—O1	110.9 (2)
N1—C1—C2	103.13 (18)	C10—C9—H9A	124.5
N1—C1—H1B	111.1	O1—C9—H9A	124.5
C2—C1—H1B	111.1	C9—C10—C11	106.0 (2)
N1—C1—H1C	111.1	C9—C10—H10A	127.0
C2—C1—H1C	111.1	C11—C10—H10A	127.0
H1B—C1—H1C	109.1	C8—C11—C10	107.0 (2)
N2—C2—C1	103.57 (19)	C8—C11—H11A	126.5
N2—C2—H2A	111.0	C10—C11—H11A	126.5
C1—C2—H2A	111.0	O4—C12—C6	124.7 (2)
N2—C2—H2B	111.0	O4—C12—C13	116.65 (18)
C1—C2—H2B	111.0	C6—C12—C13	118.62 (18)
H2A—C2—H2B	109.0	C18—C13—C14	118.5 (2)
N1—C3—N2	108.79 (19)	C18—C13—C12	120.01 (19)
N1—C3—C6	127.7 (2)	C14—C13—C12	121.3 (2)
N2—C3—C6	123.53 (19)	C15—C14—C13	120.8 (2)
N2—C7—C8	112.63 (18)	C15—C14—H14A	119.6
N2—C7—C4	109.83 (17)	C13—C14—H14A	119.6
C8—C7—C4	109.13 (18)	C16—C15—C14	120.1 (2)
N2—C7—H7A	108.4	C16—C15—H15A	120.0
C8—C7—H7A	108.4	C14—C15—H15A	120.0
C4—C7—H7A	108.4	C17—C16—C15	119.8 (2)
N3—C4—C5	109.63 (18)	C17—C16—H16A	120.1
N3—C4—C7	110.51 (18)	C15—C16—H16A	120.1
C5—C4—C7	111.60 (18)	C16—C17—C18	120.2 (2)
N3—C4—H4A	108.3	C16—C17—H17A	119.9
C5—C4—H4A	108.3	C18—C17—H17A	119.9
C7—C4—H4A	108.3	C17—C18—C13	120.5 (2)
C6—C5—C4	111.62 (18)	C17—C18—H18A	119.8
C6—C5—H5A	109.3	C13—C18—H18A	119.8
C4—C5—H5A	109.3		
			
C3—N1—C1—C2	−4.9 (3)	C4—C5—C6—C12	148.1 (2)
C3—N2—C2—C1	3.0 (3)	C9—O1—C8—C11	0.8 (3)
C7—N2—C2—C1	174.5 (2)	C9—O1—C8—C7	178.42 (19)
N1—C1—C2—N2	1.0 (3)	N2—C7—C8—C11	−163.8 (2)
C1—N1—C3—N2	6.9 (3)	C4—C7—C8—C11	74.0 (3)
C1—N1—C3—C6	−171.3 (2)	N2—C7—C8—O1	19.2 (3)
C2—N2—C3—N1	−6.2 (3)	C4—C7—C8—O1	−103.0 (2)
C7—N2—C3—N1	−177.64 (19)	C8—O1—C9—C10	−0.9 (3)
C2—N2—C3—C6	172.1 (2)	O1—C9—C10—C11	0.6 (3)
C7—N2—C3—C6	0.7 (3)	O1—C8—C11—C10	−0.4 (3)
C3—N2—C7—C8	−97.8 (2)	C7—C8—C11—C10	−177.5 (2)
C2—N2—C7—C8	91.7 (3)	C9—C10—C11—C8	−0.1 (3)
C3—N2—C7—C4	24.0 (3)	C3—C6—C12—O4	2.1 (3)
C2—N2—C7—C4	−146.4 (2)	C5—C6—C12—O4	−174.3 (2)
O2—N3—C4—C5	−58.9 (3)	C3—C6—C12—C13	−178.84 (19)
O3—N3—C4—C5	119.4 (3)	C5—C6—C12—C13	4.7 (3)
O2—N3—C4—C7	177.6 (2)	O4—C12—C13—C18	55.0 (3)
O3—N3—C4—C7	−4.0 (3)	C6—C12—C13—C18	−124.1 (2)
N2—C7—C4—N3	72.3 (2)	O4—C12—C13—C14	−120.7 (2)
C8—C7—C4—N3	−163.80 (17)	C6—C12—C13—C14	60.2 (3)
N2—C7—C4—C5	−50.0 (2)	C18—C13—C14—C15	−1.1 (3)
C8—C7—C4—C5	73.9 (2)	C12—C13—C14—C15	174.7 (2)
N3—C4—C5—C6	−69.8 (2)	C13—C14—C15—C16	1.9 (4)
C7—C4—C5—C6	53.0 (2)	C14—C15—C16—C17	−0.6 (4)
N1—C3—C6—C12	2.7 (3)	C15—C16—C17—C18	−1.6 (4)
N2—C3—C6—C12	−175.3 (2)	C16—C17—C18—C13	2.5 (3)
N1—C3—C6—C5	179.4 (2)	C14—C13—C18—C17	−1.1 (3)
N2—C3—C6—C5	1.4 (3)	C12—C13—C18—C17	−176.9 (2)
C4—C5—C6—C3	−28.5 (3)		

### Hydrogen-bond geometry (Å, °)

**Table:**

*D*—H···*A*	*D*—H	H···*A*	*D*···*A*	*D*—H···*A*
N1—H1A···O4	0.88	2.17	2.714 (2)	119
N1—H1A···O4^i^	0.88	2.40	3.025 (3)	128

Symmetry codes: (i) −*x*, *y*, −*z*+3/2.
